# Phages for One Health: regulatory and product life cycle considerations

**DOI:** 10.1099/mic.0.001626

**Published:** 2026-04-28

**Authors:** Liberty Duignan, Evelien M. Adriaenssens, Rosanna C. T. Wright, Annette Sansom, Ian MacLeod, Tobi E. Nagel, Joshua D. Jones, Joanne L. Fothergill, Carmen H. Coxon

**Affiliations:** 1 Clinical Infection, Microbiology and Immunology, University of Liverpool, Ronald Ross Building, 8 West Derby St, L69 7BE, Liverpool, UK; 2 Biologics, CPI, Central Park, The Nigel Perry Building, 1 Union St, Darlington DL1 1GL, UK; 3 Quadram Institute Bioscience, Norwich Research Park, Colney, Norwich NR4 7UQ, UK; 4 Faculty of Biology, Medicine and Health, University of Manchester, Oxford Road, Manchester, M13 9PT, UK; 5 Campden BRI, Station Road, Chipping Campden, GL55 6LD, UK; 6 JA Kemp LLP, 80 Turnmill Street, London, EC1M 5QU, UK; 7 Phages for Global Health7 383 62 Street, Oakland, CA 94618, USA; 8 UK Health Security Agency, Manor Farm Road, Porton Down, Salisbury, Wiltshire, SP4 0JG, UK; 9 MHRA Science Campus, Blanche Lane, South Mimms, Potters Bar, Hertfordshire, EN6 3QG, UK

**Keywords:** bacteriophage, food, One Health, regulation, therapeutics, veterinary

## Abstract

There is growing interest in bacteriophage (phage) technologies across the One Health spectrum. The UK Parliament’s Science, Technology and Innovation Select Committee recently published the results of its inquiry into ‘the antimicrobial potential of bacteriophages’ with recommendations on clarity on regulatory matters to support research and innovation. Products developed for different sectors will have different regulatory requirements. Here, we discuss how phage technology is applied across human, veterinary and food sectors, highlighting key regulatory considerations and the technical challenges that must be addressed to assure the quality, safety and efficacy of phage products. We also highlight the potential impact of phages in areas where they are most needed (low- and middle-income countries).

## Introduction

Bacteriophage (phage) therapy is the use of bacterial viruses as a medicine to lyse bacteria and treat bacterial infection. This approach is experiencing a revival, largely driven by the urgent need to find solutions to address the growing impending antimicrobial resistance (AMR) crisis in human medicine [1]. Beyond this, phages offer additional opportunities to control microbial growth and contamination across the food and veterinary sectors, offering an alternative to antibiotics, biocides and preservatives [2,3]. Georgia has been using phages therapeutically for over a century, and its Eliava Institute has treated tens of thousands of patients [4]. Indeed, countries all over the world are now exploring phage adoption across the One Health spectrum. Examples range from the Australia Phage Network [5], which aims to deliver country-wide phage therapy for people with antibiotic-resistant infections, to national phage stakeholder symposia that have been organized in Kenya, Malaysia and Uganda, the latter two supported by the Global Antimicrobial Resistance Innovation Fund (GAMRIF) [6].

Antibiotics are extremely important drugs that have enabled vast improvements to human health and quality of life since their introduction – childbirth, surgery, immunosuppression-related therapies for cancer and transplantation would all have higher mortality without antibiotics [7]. Yet antibiotics have downsides – toxicity from prolonged use and microbiome disruption have serious consequences and long-term health implications. Narrow-spectrum antibiotics can spare host microbiome and are less likely to drive antibiotic resistance, but this is a challenge from a drug development perspective, and due to a general loss of pharma interest in antibiotic development, these products are not widely in place [8]. Phage therapy can deliver highly targeted antimicrobial activity and can be used for both common infections and rare ones, enabling the development of products ranging from off-the-shelf to highly personalized. Phages exist everywhere bacteria exist, and it is often a matter of simply finding that phage. Rather than 2–3 years of target and drug discovery and hit-to-lead development, a phage can be found, amplified and formulated in a matter of days to weeks [9].

In 2023, an inquiry was launched by the UK’s House of Commons Science, Innovation and Technology Committee (SITC) to understand the ‘antimicrobial potential of bacteriophages’ [10], and a number of recommendations were made. Clarification around how phage products will be regulated was highlighted as an important step in enabling phage therapy. This is interesting because from a regulatory standpoint, there is a clear regulatory framework for phages – in the human and animal sector, phages are broadly classed as biological medicines, for which regulatory guidance exists. The uncertainty amongst innovators relating to regulation indicates that the available regulatory guidance is either not easily accessible or understandable. To provide the clarity that was needed, the MHRA has compiled a document bringing together all the relevant regulatory information pertaining to phage therapeutic products (PTPs) in the human sector in one place, detailing which guidance documents apply. Regulatory guidance can be inaccessible to those not familiar with regulatory language or lacking prior regulatory experience, so ensure that the information can be interpreted by those who need it. The MHRA has developed a second document to assist phage innovators in understanding the guidance. This documentation, the first of its kind, was developed in collaboration with the Innovate UK Phage Innovation Network for publication in an academic journal where those who need it will find it.

Phage products have the potential to tackle pathogens and AMR across One Health, and the regulatory requirements across food, veterinary and human sectors will be different. Furthermore, innovative products such as genetically modified phages may touch on more than one regulatory agency [e.g. in the UK, an engineered product will be evaluated by the MHRA or Veterinary Medicines Directorate (VMD) as a medicine, and Defra for its potential environmental impact], and it is important that innovators are clear on where these regulatory touch points are. An open and ongoing dialogue between innovators and regulators is essential to ensure that regulatory requirements are aligned with the technology. Here, we discuss some of the technical challenges involved in meeting the regulatory requirements for phages and how we as a community can address them. We also discuss opportunities and regulatory challenges for phages across other parts of the One Health spectrum, as well as international challenges related to implementing new technologies in low- and middle-income countries (LMICs). This publication reflects the views of subject matter experts and the MHRA Science Campus and should not be construed to represent the official view of any given regulatory authority – it is important that you always consult the regulatory authority with whom you intend to file for clarity and advise.

## Regulatory activity in the phage medicinal product space

As a starting point, phage medicinal products for humans and animals are classed as biologics, and regulatory guidance to support the development can be found at the International Council for Harmonisation of Technical Requirements for Pharmaceuticals for Human Use (ICH) [11] or International Cooperation on Harmonisation of Technical Requirements for Registration of Veterinary Medicinal Products (VICH) [12], respectively. These guidance documents help product developers identify what evidence/data needs to be provided for regulators to review to aid decisions on whether a license can be granted.

**Fig. 1.**
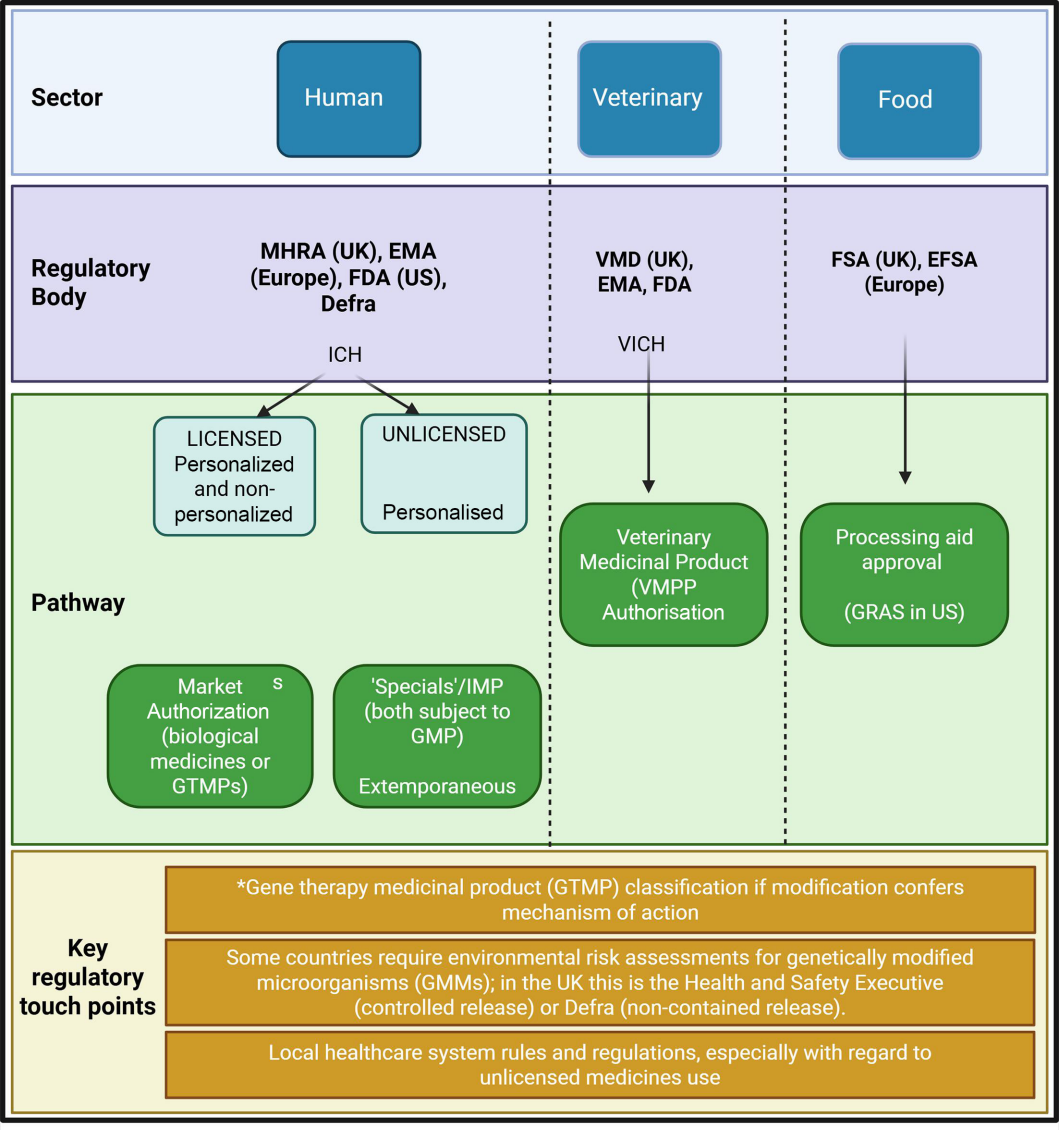
Current regulatory pathways for phage therapeutics across sectors. MHRA, Medicines and Healthcare products Regulatory Agency; EMA, European Medicines Agency; FDA, US Food and Drug Administration; Defra, Department for Environment, Food and Rural Affairs; FSA, Food Standards Agency; EFSA, European Food Standards Agency; ICH, International Council for Harmonisation of Technical Requirements for Pharmaceuticals for Human Use; VICH, International Cooperation on Harmonisation of Technical Requirements for Registration of Veterinary Medicinal Products; GTMP, gene therapy medicinal product; GMP, good manufacturing practice; VMPP, veterinary medicinal product; GRAS, generally recognized as safe.

Whilst phages broadly fall within the biological medicines class, some PTPs will be more akin to other types of medical products (e.g. gene therapy medicinal products) from a regulatory standpoint. For example, phage cocktail products (a product that contains two or more different phages rather than a single phage) may need revising over time to maintain efficacy against circulating strains. There are already examples of medicinal products that are updated over time and there is a mechanism through which this can be done where appropriate, but the exact route phage cocktail updates will take hasyet to be determined; developers will need to speak to the regulator they are engaging with to determine the best way forward. Changes to phage cocktail composition was broached in the European Medicines Agency ‘Quality, safety and efficacy of bacteriophages as veterinary medicines – Scientific guidance’ [13], published in 2024 and the recent EMA Guideline on quality aspects of phage therapy medicinal products contains a section on ‘Composition of the finished product and post-marketing authorisation change of active substance’ highlighting that regulators are aware of the intention of product developers to seek product updates. Phages have the potential for large-scale use in the population (off-the-shelf products), or as personalized medicines. Phages are not the first type of personalized medicine to seek regulatory approval, and there are regulatory mechanisms in place that permit the production of small-scale, personalized medicines. Early communication with regulators would ensure a full understanding of how the phage product being developed will be reviewed and what regulatory path should be taken (Fig. 1).

Due to antimicrobial development shifting from big pharma to academic or Small and Medium-Sized Enterprise (SME) entities, there is a lack of knowledge or experience; the existence and location of relevant guidance may not be known. Furthermore, language and terminology used by regulators can also be a major barrier because the information is written for regulatory experts, not academics or SME professionals. Industry tends to have established, open dialogue with regulators due to years of engagement and information sharing, but this is not the case for phage innovators in the academic and SME sector; there has historically been limited communication in either direction, leading to a lack of awareness of each other’s activities in this space. The MHRA phage guidance aims to provide some clarity and direction for innovators in the human health space, but regulation can span more than one regulator and expectations across One Health regulators are different. In the UK, the VMD is currently considering ‘requirements of a fit-for-purpose regulatory framework for phage-based medicines [including] guidance for the manufacturers and developers of phage-based VMPs’. Clarity is needed to highlight who does what in the regulatory space across One Health and how to interpret the requirements. Below, we explain the broad regulatory terms and expectations of phage products and highlight the differences between licensed and unlicensed use. Intellectual property and global challenges to phage product access and adoption are discussed below and summarized in Table 1.

**Table 1.** Development gaps and needs in phage therapeutics

Development gap	Current barrier	Needed solution
Standardized phage product testing	Lack of harmonized assays and reference material	Global standards for *in vitro* and *in vivo* phage activity testing and appropriate and reference material to validate assays
Global strain surveillance (esp. LMICs)	Fragmented, regional strain data and appropriate phages mapped	International phage-bacteria strain surveillance networks
Phage genome characterization standards	Poor sequence database quality	WHO-endorsed phage reference genome materials
Reference materials to validate and standardize methodologies	No way to assess and assure intra- and inter-lab assay performance or validate methods	Physical reference material, e.g. commercial, in-house, WHO-endorsed international standard
Access pathways in LMICs	LMIC regulators dependent on HIC approvals	Collaborative, region-specific regulatory capacity building
IP and commercialization misconceptions	Fear of weak patentability deterring investors	Clear IP strategies, subscription models, education of stakeholders

LMIC - Low and Middle Income Country.

## Quality, safety and efficacy – regulatory cornerstones

Regulation is there to ensure that users have access to products that are well characterized and that the benefits outweigh the risks. Risk itself is not a barrier to regulatory approval, provided the risk is fully understood, controlled and mitigated where possible. Identifying risk relies on a comprehensive understanding of the product at the physical level (its qualities) and understanding how those physical qualities relate to its activity (efficacy), as well as identifying how they may pose potential risk (safety). Risk(s) must be minimized wherever possible. It is accepted that medicinal products often come with risk(s), but these should be reduced or managed.

Regulators need to see evidence that developers have a thorough understanding of what their product looks like at the biochemical/biophysical level (its ‘qualities’) and how any manufacturing and post-manufacturing processes affect those attributes (qualities) (see Alseth *et al.* [14] for an example). Product developers also need to understand what risk (to safety and efficacy) is imparted by changes in quality and make sure that risk is minimized, controlled and mitigated wherever possible. For PTPs, considerations of quality, safety and efficacy and regulatory considerations are a relatively new area.

## Phage banks and product characterization

For many biological medicines, quality starts with banked cell stocks, and this is also true for PTPs. Banked phages and their host bacterial strains must be well characterized with metrics in place to measure and monitor quality attributes throughout the product journey including scale up, manufacture, storage, shipping and other post-manufacturing processes. In line with regulatory requirements, and as outlined in the Interpretation Guidance for MHRA Regulatory Considerations for Phage Therapeutic Products [15], banked phages and host strains used in MAAs must undergo regular requalification. Working cell bank requalification should be performed on an annual basis and for master cell banks every 3–5 years. Full genome annotation of both phage and host should be provided to the regulator with details of any genes that pose a risk, e.g. lysogeny genes (genes within a prophage that allow it to switch to the lytic life cycle therefore giving a risk of lysogenic phage contamination within the phage product), toxins, antibiotic and multidrug resistance genes, putative virulence factors, mobile gene elements and prophages. It should be noted that whilst many regulators will likely prefer phages and their host to be free of undesirable elements, it is acknowledged that this is not always possible, in which case these elements should be fully characterized and the risk fully evaluated with mitigations in place where possible. Regulators may have different opinions on risk/benefit, so it is always advised to check with the regulator in the country where registration is planned. Where phages have been sourced *de novo* from the environment (i.e. not from a formal bank), it is important that good manufacturing practice (GMP) is applied as early as possible and that all materials used to grow, manufacture or store the phage(s) and host(s) are fully traceable and characterized. The importance of ensuring phage banks are compliant with international standards cannot be overstated, especially if we are to share our banks globally [16].

### Genetic characterization

Sequencing is an excellent approach for fully characterizing phages and hosts at the genetic level and for tracing them throughout the ‘product life cycle’. As phages can mutate during adaptation to a host or between batches, resequencing them routinely is recommended to identify the acceptable genetic variability of the product. Many open source pipelines are available to support analysis; however, it is widely acknowledged that the quality of phage sequence databases is limited and the breadth of phage genomic data is lacking. This can also impact patenting, as discussed below. Reference materials should be used to validate methodologies wherever possible. The value of such reference materials for sequencing and bioinformatics has recently been demonstrated for the microbiome field. The first World Health Organization (WHO)-endorsed whole cell [17] and DNA standards [18] for the microbiome have given researchers the ability to identify and eliminate experimental bias from their processes. The MHRA is currently evaluating the feasibility of a genomic standard for phage that will provide innovators with a way to validate methodological approaches and give regulators confidence in the validity of those approaches.

### Phage taxonomy

Phage taxonomy is a fast, evolving area. The taxon to which a phage, or indeed any micro-organism, is assigned can change, and such changes require an update to market authorizations. Similarly, if the taxonomy of a phage that is the active pharmaceutical ingredient of a medicinal product changes, a variation to the marketing authorization [19] is required. It is therefore important to know how a phage is classified and if it changes as this is an evolving area. Phage taxonomy falls under the remit of the International Committee on Taxonomy of Viruses (ICTV) and is managed by the Bacterial Viruses Subcommittee. The ICTV evaluates, approves and ratifies the creation of new taxa, their demarcation criteria and their nomenclature. This evaluation process is organized around the submission of taxonomy proposals by the general virology community to specialized Study Groups for assessment, collection and quality control of proposals by the Subcommittee Chair, an approval vote by the Executive Committee and a ratification vote by the ICTV membership [2]. Following the yearly ratification vote, a new taxonomy is published on the ICTV website (https://ictv.global) and available as a set of spreadsheets (Master Species List and Virus Metadata Resource). Virus taxonomy is organized in a 15-rank hierarchical structure reflecting the evolutionary history of viruses it contains, with currently six realms further subdivided into subrealm, kingdom, subkingdom, phylum, subphylum, class, subclass, order, suborder, family, subfamily, genus, subgenus and species ranks [3,4]. Phages are currently classified into five realms (*Duplodnaviria*, *Monodnaviria*, *Varidnaviria*, *Singelaviria* and *Riboviria*) and two unclassified families (MSL40, March 2025 ratification vote). The vast majority of isolated phages are assigned to the realm *Duplodnaviria* and the class *Caudoviricetes*, where they are further subdivided into 71 families, 1,633 genera and 5,766 species [20]. As of 2022, phage taxonomy is only based on genomics, no longer morphology, resulting in the abolishment of the decades-old families *Myoviridae*/*Podoviridae*/*Siphoviridae* and the uplift of the order Caudovirales to the class Caudoviricetes [6]. As a result, many of the phage species, genera and subfamilies for which new families were not defined yet are currently listed as unclassified within the class Caudoviricetes. It is not anticipated that this would preclude MAA approval, so long as sufficient product characterization was done and all risks were evaluated. The lowest rank currently in use by the ICTV is the species rank. Each species has an exemplar virus (or in this case phage) associated with it for which the genome is available as an annotated record from GenBank through submission to one of the INSDC databases (International Nucleotide Sequence Database Collaboration) [7,10]. Whilst many species only have one known isolate, there are several species which contain multiple phages. To make the distinction between the species (a monophyletic group of viruses within a set of demarcation criteria, i.e. a human-made grouping) and a phage (a physical entity), the ICTV is using a binomial nomenclature system for species (e.g. *Kuttervirus ViI*) and a common naming system for members of the species (e.g. *Salmonella* phage Vi01), see also [11,12].

The ICTV makes no assessment on whether a phage is considered a ‘new phage’ for product development or regulatory purposes and will only assess whether – based on the full genome sequence – it should be assigned to a different taxon. For phage in the class Caudoviricetes, phages belong to the same species if their genomes across the genome length have more than 95% nucleotide identity. From a regulatory perspective, it is expected that product developers will have to justify the acceptable range of nucleotide divergence on a product-by-product or species-by-species basis.

### Biological activity

Understanding the host range of a phage and its impact on host viability is important. Classically, phage activity can be investigated using agar-based assays, such as plaque assays, spot tests or direct plating (these bacteria-to-phage susceptibility tests are commonly called ‘phagograms’). Phage activity against planktonic bacterial cultures can also be investigated by using spectrophotometry to assess cell density or respiratory activity. Unfortunately, phagograms are currently poorly standardized. Moreover, not every host–phage pair behaves the same in these assays, and not every phage–host pair behaves the same across different assay platforms [21]. Due to the lack of consistency between methodologies, the use of controls to provide confidence of assay performance, reproducibility and sensitivity should be used wherever possible and will give regulators confidence in the methodologies and their suitability to characterize the product (see also ICH Q6B [22]). It is important to note that regulators are not prescriptive on which methodologies should be used. For some product types, there are established methods developers are encouraged to use as they are well understood and characterized (e.g. pharmacopeial methods); however, especially in the case of novel medicines, new methodologies may be needed which regulators do not know about or have experience with. It is therefore up to the developer to decide which methodologies are best and ensure they have provided the regulator with enough information to assess it. Again, the inclusion and appropriate use of controls and reference reagents is an essential part of assay validation and assurance.

To improve the biological activity of phages, they can be serially cultured on specific bacterial host strains to select for naturally arising variants that improve efficacy, without introducing foreign genetic material. This can be an option when producing a phage for a named patient in a personalized medicine approach by using the patient’s own strain. It is recommended that phages and production hosts are genetically characterized to identify any potential risks to the patient due to the presence of deleterious genetic elements – the decision to use the phage will be based on the risk posed by the phage/host and the patient need.

Another aspect of phages is their specificity. Although highly selective for their targets, fully characterizing host range is an important step in PTP development, especially as periodic PTP variations will be required to keep pace with bacterial evolution. Well-curated bacterial collections should be tested for phage–host range, and such a collection should reflect the overall population structure of the bacterial phage target in terms of genotype and phenotype. Such data would ensure that phage therapy is correctly utilized, but this approach also requires suitable tests and diagnostics to determine susceptibility.

### Purities, impurities and contaminants

Impurities can be process- or product-related. The presence of an impurity or contaminant is not necessarily a problem if it is known, characterized and either shown not to constitute a risk or is mitigated. Fermentation is used widely to manufacture biological medicines, and assays for known contaminants are generally well defined. A risk-based approach should be used to evaluate impurities and contaminants. With regard to PTPs, examples of impurities could be pyrogens, toxins, host cell proteins and host cell DNA. Innovators developing a PTP will need to have assays in place to detect and monitor these contaminants. Again, we recommend reading the MHRA *Regulatory considerations for therapeutic use of bacteriophages in the UK* and the accompanying *Interpretation guidance for MHRA regulatory considerations for phage therapeutic products*, which are important reference documents for this. The importance of having validated methods to measure quality attributes of phage products is paramount so that the impact of changes to manufacturing processes or scale can be assessed.

## Guidance on product development and post-market vigilance of PTPs

More information about this subject in the context of phage medicinal products can be found in the MHRA *Regulatory considerations for therapeutic use of bacteriophages in the UK* and the accompanying [15]. *Interpretation guidance for MHRA regulatory considerations for phage therapeutic products* [23]. MAAs for PTPs for humans should conform to the common technical document (CTD) format published on the ICH website [24]. For veterinary medicines, some information on standardized document provision can be found on the EMA website and the UK VMD also provides guidance on marketing authorizations for veterinary medicines [25,27].

## Commercialization and intellectual property challenges

Commercialization and protecting intellectual property (IP) are cornerstones of product development. The applicability of phage to traditional IP routes has also been identified as a challenge. Paragraph 30 of the SITC report states ‘…natural phages maybe difficult to patent, though unique combinations of phages and engineered phages might be more amenable to intellectual property protection. This has been seen as a major disincentive for pharmaceutical companies to invest in phage manufacturing, and also in clinical trials with an uncertain return on investments’.

Despite potential challenges presented by the complexity of phages, patent protection can be obtained for various aspects of phage products and associated methods, including products containing naturally occurring and previously described phages. The number of patent applications directed to phage has been steadily increasing over the last 20 years. A simple analysis of international and European patent applications classified in CPC category A61K35/76 (bacteriophages) and including ‘phage’ in the title has increased from 1 to 2 per year in the early 2000s to 10–15 per year in the last 5 years. New companies developing phage products and therapies should consider their patent strategy as early as possible to ensure no opportunities are lost by first publishing their work. This ensures robust patent portfolios that will be attractive to investors. It is also essential that those supporting new companies developing phage therapies, e.g. investors and university technology transfer specialists, dispel negative misconceptions regarding the patent application process and encourage scientists to develop their patent strategy.

Many common assumptions that patent protection will be difficult to obtain for phage products stem from a 2013 decision by the US Supreme Court in Association for Molecular Pathology v. Myriad Genetics, Inc. [28]. In this case, the Court ruled that naturally occurring DNA sequences cannot be patented simply because they have been isolated, as they are considered products of nature. This decision initially led to a restrictive interpretation at the US Patent and Trademark Office (USPTO), marking it more difficult to obtain patents for various biological inventions, including phages. Given the significance of the US market, this had a broader impact and a dampening effect on perceptions around the patentability of phage-based products globally, even though patent laws and practices have since evolved for biological inventions.

However, in the years since the US Supreme Court’s decision, other jurisdictions have maintained patent systems that recognize a very broad range of biological inventions. In addition, today, the USPTO is generally more pragmatic, and patent attorneys are familiar with presenting biological inventions in a way that ensures they are not considered to be directed to a law of nature or a natural phenomenon, including by clearly presenting and claiming the inventive concept that provides the practical application, which is patentable [29]. Whilst it still remains difficult to obtain patent protection for many diagnostic assays in the USA, the same is not true for phage products and therapies.

If a phage is identified to be useful in the treatment of a particular bacterial infection, that development is potentially patentable as a method of treatment (USA) or as the phage for use in the new treatment (other jurisdictions). Furthermore, patent offices around the world routinely grant patents for many different aspects of new treatments to incentivize the development of new treatments, particularly those using known agents. Modified and engineered phages can be claimed directly, with the breadth of protection granted dependent on how similar the modified phage is to a known phage. Any new modification is potentially patentable, including deletions and minimal substitutions. The complexity of phage products and their manufacture provides yet more opportunities for obtaining valuable patents because each innovation to develop a feasible product may be protected. This includes, for example, aspects such as formulations, doses, administration regimens and patient sub-groups, as well as discovery platforms (screening methods), manufacturing, purification methods and cocktail compositions. Again, this also applies to known phage, so new combinations of known phages may be patentable, and new approaches to formulating, purifying and screening known phages may also be patentable.

The complexity of phage products can present challenges in relation to building a patent portfolio, but these are not insurmountable. It is necessary to define a new phage, or new phage product, precisely, such that patent claims do not extend to known phage or products, whilst, at the same time, ensuring that the patent claims are broad enough to cover variations that competitors may attempt to market, or those required during product development. This is particularly challenging for phage products because phage taxonomy is poorly characterized, despite the ongoing efforts described above. Therefore, it is much harder to claim a precisely defined group of phages compared to, e.g. bacteria, which are often claimed at the level of strain, species or genus.

Accordingly, it is often necessary to claim a new phage or phage product with reference to a deposit with a depositary authority. This is commonly done with bacterial products, but patent claims referring to specific deposits are very narrow, and the exact scope of claims referring to deposited bacterial strains and how they might be infringed is not clear [30]. These issues are more complex for phage products because the sequence of phage genomes changes much more rapidly, so a patent claim to a deposited phage may quickly become irrelevant. Therefore, it is often appropriate to claim phage with reference to their genome sequence, ideally allowing for significant sequence variation, but this approach is complicated by challenges in phage sequence assembly and annotation, often making it difficult for patent applicants and examiners to compare whole-genome sequences in a reliable and consistent way. Many patent applicants and examiners are familiar with using nucleotide and polypeptide sequences in patent claims for more established products such as vaccines and antibody therapeutics, but using phage sequences in the same way may not be appropriate or ideal.

Going forward, approaches for drafting patent claims directed to phage and phage products should be flexible and combine genomic, phenotypic and functional metrics/characteristics. This will allow a patent applicant to precisely define their phage product as required and address any objections or prior art documents that might be raised by patent examiners during examination. It will also ensure that the patent claims continue to cover the product as it progresses through development and ideally similar products that competitors may be tempted to develop.

Furthermore, innovative approaches to the adoption of phage therapies will provide assurances to investors and increase their attractiveness. In particular, the UK Government has recently introduced a subscription model for procuring antimicrobial products, and the same or a similar model could be very effective for phage products. The subscription model provides a contract whereby the UK National Health Service (NHS) pays companies a fixed annual fee for antimicrobials based on the value to the NHS of the product’s availability, rather than volumes used. This approach would encourage companies to invest in the development of phage products (and other novel antimicrobials) because it would provide a predictable revenue stream, even for products that are used only sparingly.

Patent protection and an established subscription model for procurement would enable companies to have confidence that their investment in phage therapies is sustainable. If the subscription model is applied to phage therapies, it will still be important for companies to build robust patent portfolios because the UK subscription model can provide contract extensions and ongoing annual fees that last up to the expiry of patent exclusivity for a particular antimicrobial product [31]. Therefore, consistent with conventional medicines, there is a clear incentive to make sure that all technical developments that are made throughout a product’s path to patients are reviewed to identify innovations that may be patentable.

In summary, phage product development will require significant investment which, like many fields, will be very hard to secure in the absence of a robust patent portfolio and in the face of perceived challenges, which are often cited as a barrier to the development of phage therapies [32,33]. Much of the demanding work and investment required to develop phage therapies can potentially provide valuable patents provided the relevant opportunities are seized and patent applications filed at the correct times; an effective patent portfolio strategy will follow the development of a product through its progression to patients and will also be a valuable asset to support that progression.

## Towards clinical use

It is for the innovator, not the regulator, to design preclinical and clinical work packages. It is very important to think carefully about what outcomes you will measure and how the product will be introduced into real-world use in a healthcare setting. It is also worth noting that the needs of investors and the needs of the patients may not always align. For example, uncomplicated urinary tract infections have good preclinical models that translate well to humans, and there is a significant market to assure revenue generation through sales. Investors want to see a return on their investment, and targeting a highly prevalent indication supported by good *in vivo* models is far less risky than a rare clinical presentation that is hard to recruit for a clinical trial and lacks appropriate preclinical models. Indications of greater unmet need are more challenging for developers because whilst they may ultimately provide greater therapeutic impact, it is less attractive to investors and harder to build the evidence case for efficacy.

In the UK, the MHRA’s responsibility is to evaluate trial design for safety and approve the study in parallel with the NHS and designated research and ethics committee, who validate the ethical aspects of the study. An in-depth discussion of pre-clinical and clinical work packages appropriate for PTPs is beyond the scope of this manuscript [34], but innovators should ensure they keep a focus on quality, safety and efficacy throughout.

## Regulatory status and legal basis for supply (licensed and unlicensed)

The MHRA *Regulatory considerations for therapeutic use of bacteriophages in the UK* details the various regulatory frameworks that may apply to phages. This includes licensed and unlicensed products, and it is recommended that product developers in the human healthcare product sector use this document to guide them through the regulatory landscape. It is also important that developers are aware of additional legal frameworks in the context of producing and supplying medicinal products (e.g. good manufacturing, distribution and pharmacovigilance practice).

### Licensed products

In the UK, the MHRA regulates medicines, blood products and medical devices, and those wishing to sell medicinal products on the UK market must prepare an MAA for the regulator to review, covering safety, quality and efficacy. PTPs developed for use in the UK veterinary sector are regulated by the VMD. Phage products developed for use in the UK food sector will be regulated by the Food Standards Agency (FSA) or Food Standards Scotland. Regulation of phages in the food sector will be discussed in more detail below. In the UK, genetically modified phages will also be subject to regulation by the Department for Environment, Food and Rural Affairs (Defra) and/or the Health and Safety Executive (HSE). Defra regulates uncontained risks (wider environment) and HSE regulates contained risks (e.g. hospitals or home environment).

At the global level, there will be similarities and differences in how phage products, including genetically modified (GM) phage products, will be regulated; not all regulators are the same.

When deciding which market to enter, it is important that care is taken to identify the specific requirements of the national regulatory authority (NRA) in that country. Many countries use the ICH CTD as a template, but not all. Indeed, in Southeast Asia, a modified ICH CTD is used, and there are many examples of country-specific considerations that need to be factored into preclinical and clinical packages.

### Phages as unlicensed medicines

Unlicensed medicines are those without a marketing authorization granted by the NRA. In the UK, this includes ‘specials’, imports and investigational medicinal products. Phages can be used as an unlicensed medicine where licensed products are unable to meet the clinical needs of a patient and are referred to as phages for compassionate use (a more common term in the academic literature). Unlicensed medicines produced in the UK must be made in accordance with GMP, whilst non-GMP unlicensed medicines may be imported provided they meet equivalent quality standards as assessed by the MHRA and NHS. Note that using unlicensed medicinal products has regulatory touchpoints across the healthcare system and that access to unlicensed medicines does not sit entirely with the medicines regulator.

### Engineered phages

Genetic engineering provides not only a route to more comprehensive IP protection but also offers the potential for enhanced attributes including increased host range, improved quality, safety, efficacy, stability and other advantageous properties. However, the use of genetic engineering does, in some cases, affect how they are regulated. In the EU, the definition of a gene therapy, as given in SI 2019 775, which amends the Human Medicines Regulations of 2012, states ‘A “gene therapy medicinal product” is a biological medicinal product which has the following characteristics: (a) it contains an active substance which contains or consists of a recombinant nucleic acid used in or administered to human beings with a view to regulating, repairing, replacing, adding or deleting a genetic sequence; and (b) its therapeutic, prophylactic or diagnostic effect relates directly to the recombinant nucleic acid sequence it contains, or to the product of genetic expression of this sequence’.

As such, in the EU and UK, phages carrying genetic modifications that relate to the mechanism of action are classed as GTMPs. Examples could be the inclusion of a payload (e.g. a protein toxic to bacteria) essential to the mechanism of action. Note that modifications pertaining to improved quality or safety, such as removal of toxins or other deleterious genetic elements, are not related to mechanism of action and, therefore, these products remain as biologicals and not GTMPs.

In the UK, engineered phage products must also undergo environmental risk assessment. This is because phages, unlike many other therapeutics, can be released into the environment and replicate. Given the known issues of antibiotic residues contributing to increased AMR in environmental settings, similar considerations apply to the use of phages. Product developers should contact Defra and/or HSE to seek further guidance for access to the UK market or the relevant NRA.

### Patient acceptance

The development of PTPs as human medicines should be patient-centred, and care may need to be taken around clear communication with patients, especially for engineered PTPs. Phages have been used extensively in the geopolitical East, notably Georgia. Reassuringly, early work suggests that Western markets will be receptive to phages. A 2020 study from the UK, which explored the use of phage therapy for diabetic foot ulcers, found that whilst knowledge about phage was fairly low, concerns over their use as a treatment strategy were no greater than those for antibiotics [35]. A more recent 2023 study indicated UK attitudes towards phage as a therapy were broadly positive, especially when considered in the context of novel therapies and the threat of AMR [36]. A survey of UK health professionals reported that >70% of responding clinicians would consider using phage therapy for eligible patients. It has, however, been highlighted that the history of phage therapy could make it particularly susceptible to negative media coverage, which could, in turn, have a negative impact on adoption.

## Considerations for phage manufacture

Based on the use case, a wide range of phage stock manufacturing scales can be envisaged, from ~100 ml for an individual patient to ~10 l for large-scale preformulated human medicinal products and to several hundred litres for One Health and built environment applications, e.g. water treatment (to prevent cholera), decolonization of infrastructure (e.g. *Pseudomonas* in hospitals), animal feed additives (e.g. prevention of salmonella infection in chickens) or preventing food spoilage. Different regulators are responsible for products used across different sectors. It is known that the scale of manufacture, as well as the materials used, can have a significant effect on product quality, especially for biologics [37,39], and this must be monitored and controlled. For the manufacturing of phages in the UK for use as human medicinal products, they have to be manufactured to GMP standards, which are expensive to set up, and there is currently no facility set up to do this in the UK. Cocktail products could be produced as individual phage preparations mixed at the end of production, or as co-cultures. Co-cultured bacterial consortia products aimed at restoring microbiome function sometimes use this approach, but it is important to understand how dynamics of growth can alter final composition and therefore batch monitoring is essential to ensure the product is the same as the original characterized drug substance. Reference materials would support the assurance of measurement methodologies and are recommended by ICH guidance Q6B. More recently, cell-free phage production has been described and may provide a way to reduce or better control impurities and contaminants [40,43].

Operating at small production scales for named patient or compassionate use can mean producing as little as a single treatment course for one individual. The use of multiple single phages concurrently (as seen in the STAMP protocol [9]), or pharmacy-based combination into a bespoke cocktail preparation (as seen in the magistral approach [44]), provides a versatile strategy to enhance the therapeutic potential and limit the emergence of phage resistance, whilst retaining high standards of quality.

Operating at mid-term production scales introduces a different set of considerations in terms of production efficiency and adaptability, and again, ensuring product quality is essential throughout the manufacturing process. When designing products, it is important to consider the intended use of the product – not only the proposed recipient but also the location and setting. If the product is intended for use in remote areas in extreme temperatures, this must be considered in the product design and development. Further information around manufacture, stability, packaging and other considerations can be found in the MHRA guidance.

## Phages for one health

AMR is often described as a One Health issue as AMR is driven by the use of antimicrobials in diverse settings that include human, veterinary and food sectors. The SITC report recommended that ‘phage-related research and development across different sectors [should] be joined up as part of [the Department of Health and Social Care’s] overarching “One Health” approach to tackling AMR’*.* Here, we present some examples of phage use in food and veterinary sectors and the potential that phages have for preventing disease and improving human health through the control of pathogens in animals and food.

### Regulation of phages in food

In the USA, food is regulated by the FDA (Health and Human Services), the Food Safety and Inspection Service [FSIS, under the US Department of Agriculture (USDA)], who regulate meat, poultry and egg products. The Environmental Protection Agency (EPA) regulates pesticide and agrichemical use, whilst the Centre for Disease Control (CDC) monitors and investigates foodborne illnesses.

In the EU, the Farm to Fork strategy aims to ‘accelerate our transition to a sustainable food system’, and the establishment of the European Food Safety Authority (EFSA) assesses and provides information on all risks related to the entire food chain. Coordination between EU member states is essential to avoid the spread of disease across the landmass and trade routes of the EU. Member States and the European Commission (EC) use the rapid alert system for food and feed to facilitate rapid knowledge exchange and respond to health threats related to food or feed products. The Committee on the Environment, Public Health and Food Safety (ENVI) is a European Parliamentary Committee driving the move towards net zero, and it works closely with other policy makers in the EU around biodiversity and pesticides, amongst other areas. This highlights the complexity of regulating food products across the EU.

In the UK, products used in the food chain – both foodstuffs and medicines – are regulated by the FSA and the VMD. Recent changes to legislation mean that genetically modified crops and animals, where the genetic could have arisen through natural selection or traditional breeding, are no longer classed as genetically modified organisms (GMOs). This approach is now referred to as ‘precision breeding’. This term could also be used in phage production where phages are serially cultured on specific bacterial host strains to select for naturally arising variants that improve efficacy, without introducing foreign genetic material. However, when micro-organisms that are genetically modified (such as some phage products, discussed above) are not included in this reclassification, evaluation of the environmental impact of GM food and medicinal products must be considered by the Department of Environment, Food and Rural Affairs (Defra), which evaluates the impact of environmental release, and the Health and Safety Executive (HSE), which regulates contained release. In the UK, Defra and the HSE are joint competent authorities for GMO products. Clinical trials involving GMOs are classified as either ‘contained use’ or ‘deliberate release’ studies. GMOs can be contained chemically, biologically or physically, with biological containment comprising a modification that prevents the GMO from surviving outside the target organism. The HSE has published The Genetically Modified Organisms (Contained Use) Regulations 2014 [45] as well as Guidance notes for risk assessment outlined in the Genetically Modified Organisms (Contained Use) Regulations 2014 on the contained use of genetically modified micro-organisms [46]. Examples of approved applications for the controlled release of GMOs, including pharmaceutical GMOs [47], have been published on GOV.UK.

### The potential impact for phages in food

The potential of phages for biocontrol is huge and spans from farm to fork, offering innovative solutions to enhance food safety and sustainability without compromising natural quality or relying on chemical preservatives. The urgency for such technologies is underscored by the substantial burden of foodborne diseases, which, in the UK alone, were estimated to affect 2.4 million individuals annually, with societal costs reaching £9.1 billion in 2018 [48]. Also of importance is the requirement to reduce food waste, which creates significant environmental burdens. It has been estimated that in 2018, one quarter of all food produced was wasted [49]. Reduction of food spoilage micro-organisms within food systems by use of phage technologies could help address the associated environmental and economic issues.

Phage biocontrol technologies are being explored across various stages of food production and processing, demonstrating versatility and potential in enhancing microbial safety, and they have already been in use. Indeed, the FDA approved a phage cocktail against *Listeria monocytogenes* on ready-to-eat meat and poultry products in 2006 [50] although compliance with US Department of Agriculture Federal Meat Inspection Act and/or the Poultry Meat Inspection Act was required before the product could be used, highlighting again that regulation of products often involves more than one competent authority. There are several commercially available phage products licenced for use in the food production industry in some countries (including the USA, Canada, Switzerland, Australia and New Zealand). Examples of these commercial products are given in Table 2.

**Table 2.** Bacteriophage products in the food sector

Target organism	Product name	Manufacturer	Remark
*Escherichia coli* STEC	PhageGuard E™	Micreos Food Safety	Example applications include meat and poultry Regulatory claims include: USA, FDA GRAS (GRN 757)–2018 USA, USDA-approved processing aid (Directive 7120.1) (on beef carcasses, primals, subprimal cuts and trimmings) – 2018 Website
	EcoShield™ PX	Intralytix	Example applications include meat, poultry, fruits, vegetables, dairy, fish and seafood Regulatory claims include: FDA GRAS (GRN 834) Website
*Campylobacter*	CampyShield™	Intralytix	Example applications include raw red meat and poultry Regulatory claims include: FDA GRAS for direct application to raw red meat and raw poultry (GRN 966) Website
*L. monocytogenes*	PhageGuard Listex™	Micreos Food Safety	Example applications include meat, poultry, fruits, vegetables, dairy, fish and seafood Regulatory claims include: USA, FDA GRAS (GRN 528)–2014 Canada, Health Canada: Processing aid – 2011 Australia/New Zealand; FSANZ processing aid – 2012 Website
	ListShield™	Intralytix	Example applications include meat, poultry, fruits, vegetables, dairy, fish and seafood Regulatory claims include: FDA GRAS for direct application to fish and shellfish, fresh and processed fruits and vegetables and dairy products Health Canada approved for use on RTE meat, poultry, smoked salmon, fresh cut apples and long leaf lettuce Website
*Salmonella*	PhageGuard S™	Micreos Food Safety	Example applications include meat, poultry, fish and seafood, dairy, plant and plant-based products Regulatory claims include: USA, FDA GRAS (GRN 468) – 2013 Canada, Health Canada: processing aid – 2016 Website
	SalmoFresh™	Intralytix	Example applications include meat, poultry, fish and seafood, dairy, plant and plant-based products Regulatory claims include: FDA GRAS (GRN 435) for direct application onto poultry, fish and shellfish, fresh and processed fruits and vegetables Health Canada approved for use on fish shellfish, fresh and processed fruits and vegetables or on RTE poultry products prior to slicing and raw poultry before/after grinding Website
	Finalyse SAL™	Arm and Hammer	Target and reduce *Salmonella* in poultry processing (reduced percentage positives of detection of *Salmonella* in ground turkey samples from 46% to 28%) Website
*Shigella* spp*.*	ShigaShield™	Intralytix	Example applications include RTE meat, poultry, fish, shellfish, fresh and processed fruits and vegetables and dairy products including cheese Regulatory claims include: FDA GRAS (GRN 672) Website
Bacterial plant pathogens	Biolyse®	APS Biocontrol	An agricultural phage product to prevent soft rot (Biolyse® BP) in washed potato tubers; also offer a bespoke service to create unique combinations of bacteriophage to address plant pathogen diversity Website
*Xanthomonas campestris* pv*. vesicatoria* and *Pseudomonas syringae* pv. tomato	Agriphage™	OmniLytics Inc.	Directly kills the bacteria on the lesions that cause tomato and pepper bacterial black spot; can be used to replace copper-based bactericides that can build up in the soil and cause copper-based resistances Website

The application of phage therapy in the food industry is supported by a growing body of research, with studies documenting its effectiveness across various food matrices, including raw and cooked meats, dairy and animal by-products, alternative proteins, beverages, fresh produce, multi-component foods and even pet food. The phages in these products target pathogenic bacteria such as *Salmonella* spp., *L. monocytogenes, Campylobacter* spp., *Escherichia coli* O157/STEC, *Shigella* spp. and *Vibrio* spp*.* [51,52]. Whilst there have been fewer studies on phage control of spoilage bacteria, there are some examples, e.g. control of *Bacillus* spp., in fermented foods [53] and mashed potato; another study described the control of lactic acid bacteria in beer [54]. Variation between applications makes it difficult to produce robust efficacy data. In food use, the matrix and application method, stage of processing that the treatment is applied, methods of determining the effectiveness, range of strains applied and initial level [3] all impact composite data, with a wide range of outcomes reported, from 0.55 to 6 log reductions. These findings underscore the versatility and potential of phage biocontrol technologies in improving food safety, reducing food waste and contributing to the sustainability of the food industry.

#### Phages in the animal sector

In animal agriculture, phage therapy has emerged as a promising alternative to antibiotics in livestock and aquaculture, addressing pathogens that compromise animal health and food safety. Veterinary use of phages also extends to working and companion animals. For example, Intralytix in the USA has two products that target *E. coli* in pet food and on animal fur. Phage treatments developed by companies like Intralytix and Micreos target key pathogens in poultry, such as *Salmonella* sp. and *Campylobacter* spp., and in cattle, such as *E. coli* and *Staphylococcus aureus*. These interventions not only enhance animal welfare but also reduce the risk of pathogen transmission to humans through the food chain. Phage products could be used as feed additives, in which case they fall under the FSA in the UK, or veterinary medicines, which are regulated by the VMD. In August 2023, the EMA released regulatory guidance for PTPs developed as veterinary medicinal products, and a general chapter by the Quality of Medicines and HealthCare of the Council of Europe (EDQM) has recently been published in the 11th Edition of the European Pharmacopeia [55]. It is likely the UK’s VMD will follow EMA guidance in the foreseeable future and innovators should follow this guidance closely.

Another noticeable use for phages in food products is in aquaculture. Aquaculture is an important food source and economically important in many countries and has been identified as a potential route to empowering women farmers in LMICs [56]. There are phage products in use and more in development in the aquaculture space, and there is evidence that they are as effective as antibiotics in this context [57,59]. Phage products for fish and shellfish have been used commercially for ~10 years. Other aquaculture phage applications include treating *Vibrio*, *Pseudomonas* and *Aeromonas*. As highlighted in the WHO publication ‘Bacteriophages and their use in combating antimicrobial resistance’, phages can be used in place of chemicals and antibiotics.

Phages may also serve as an alternative or complementary application in aquaculture vaccination. Publications from NORM-VET [60], a programme monitoring antibiotic use and AMR in Norway, have shown that the use of antibiotics in aquaculture fell by 99.9% following peak use in 1987 due to the introduction of vaccination. However, fish vaccination is a labour-intensive process that can cause distress to the fish. Phages are now being used to treat salmon for *Yersinia ruckeri* [61] and are being trialled for *Pasteurella* strains that cause pasteurellosis [62]. Products that treat the water to remove the pathogen rather than the fish may be classified as biocontrol products rather than veterinary medicines, but it is important to confirm classifications with the relevant NRA before developing a licensing application.

#### Phages in crop production

Phage therapy also shows promise in plant agriculture, offering a natural solution to bacterial diseases affecting crops. Studies have demonstrated the effectiveness of phage cocktails against plant pathogens in potatoes, tomatoes, cherries, grapes and melons, significantly reducing disease incidence and severity [63]. For example, phage treatments have effectively targeted *Pectobacterium* sp. in potatoes, *Ralstonia solanacearum* in tomatoes and *Pseudomonas syringae* in cherries, showcasing the potential of phages in safeguarding crop health and yield [18,21]. In the UK, APS Biocontrol Ltd uses bacteriophage technology for food spoilage indications [64]. These applications not only highlight the effectiveness in reducing disease incidence but also its potential for integration into existing agricultural practices such as irrigation systems.

## Harmonizing phage regulation globally

Regulatory requirements for phage products are crystallizing in many high-income countries (HICs) with increased activity and increasing connectivity on requirements. However, there is increasing recognition that the need for, and impact of, phages will be even greater in LMICs with roughly 90% of the AMR-associated expected to occur in Africa and Asia by 2050 [65].

Furthermore, to meet pandemic preparedness ambitions and given that pathogenic bacteria can easily cross international boundaries, we need ‘free movement’ of phages between territories and, therefore, harmonized regulatory requirements on phage and host quality, safety and efficacy are needed. Scientists in LMICs have already begun initial work to develop phage products for applications across One Health sectors [66,69]. Working collectively and collaboratively will ensure there is minimal duplication of efforts and that globally, movements are in the same direction globally.

One challenge to these efforts is the differing regulatory systems across the globe. The WHO uses a global benchmarking tool to determine the functional level of regulatory systems in countries worldwide, designating official maturity levels (MLs) 1–4 [70]. Whilst most HICs function at ML 4, ~70% of LMICs in Africa function at MLs 1–2. As a result, LMICs often approve in-country use of medicinal products only after they have first been approved in HICs. Thus, products might eventually be exported from HICs to LMICs. However, the bacterial strains prevalent in different countries may vary, necessitating the use of phages that are specifically tailored to the local bacterial. The issue of how phage cocktail products can be updated as required without requiring full *de novo* marketing authorisation application to ensure timely updates and sustained product efficacy is a recurring one; given the potential for bacterial strains to spread across borders through travel, adapting phage cocktails in a timely manner to address emerging variants in both HICs and LMICs will be important. The European Medicines Agency veterinary guidance for PTPs alludes to the significance of such update mechanisms and outlines a potential and pathway for the implementation and, as mentioned above, the EMA Guideline on quality aspects of phage therapy medicinal products contains a section on ‘Composition of the finished product and post-marketing authorisation change of active substance’ to address this.

## Summary

Bacteriophage products have the potential to control pathogens as well as make significant gains in the fight against AMR across the human, veterinary and food sectors. Regulation is often seen as a barrier, but this is not the intention – regulators have a responsibility to ensure products deliver benefit to the public and patients without excessive risk. Regulatory guidance is meant to help developers understand what evidence is required for regulators to make a fair assessment, but the language and format can be impenetrable to those lacking regulatory experience. Here, we have tried to explain regulatory expectations and current limitations in a broad sense and apply that to phage products to enable developers to work with the existing guidance and frameworks. Phages have the potential to be used anywhere, if the regulatory frameworks and access pathways exist. Here, we have tried to emphasize that regulation does not only apply to obtaining a marketing authorization – regulation exists across the user (e.g. patient or food producer) and product journeys, so developers must understand this from end to end and plan for it.

In the UK, decisions around the use of medicinal products and medical devices in the healthcare system are made by the UK’s health technology assessment bodies, and a hospital trust can only be reimbursed for a drug it prescribes if it has health technology assessment (HTA) body approval. Whilst innovative medicine subscription models exist for some antimicrobials, the vast majority rely on profits from volumes sold. Therefore, a keen knowledge of the market potential and access is essential when seeking licensure in a country. Not all countries have HTAs, and most reimbursement models are based on volumes of drugs sold, not the overall value of the product to society as a whole. In addition, clinicians and patients need knowledge of new therapeutic approaches, and currently, this is minimal for phage therapy. Innovators must understand the entire product life cycle from ideation to registration and beyond; again, regulation is not restricted to product licensure – it applies to use as well.

Phages hold huge promise and appetite for this technology is accelerating, but it is important that those innovating in this space have full visibility of how that product will be used and where. Knowledge and communication, as always, are key.

## Abbreviations

AMR: antimicrobial resistance
CTD: common technical document
Defra: Department for Environment, Food and Rural Affairs
EFSA: European Food Standards Agency
EMA: European Medicines Agency
FDA: US Food and Drug Administration
FSA: Food Standards Agency
GM: genetically modified
GMO: genetically modified organism
GMP: good manufacturing practice
GRAS: generally recognized as safe
GTMP: gene therapy medicinal product
HICs: high-income countries
HSE: Health and Safety Executive
HTA: health technology assessment
ICH: International Council for Harmonsiation of Technical Requirements for Pharmaceuticals for Human Use
ICTV: International Committee on Taxonomy of Viruses
IP: intellectual property
LMICs: low- and middle-income countries
MAA: marketing authorization application
MHRA: Medicines and Healthcare Products Regulatory Agency
ML: maturity level
NHS: National Health Service
NRA: national regulatory authority
PTP: phage therapeutic product
SITC: Science, Innovation and Technology Committee
SME: Small and Medium-Sized Enterprise
USPTO: US Patent and Trademark Office
VICH: International Cooperation on Harmonisation of Technical Requirements for Pharmaceuticals for Human Use
VMD: Veterinary Medicines Directorate
WHO: World Health Organization
